# Salinity and Temperature Influence Growth and Pigment Production in the Marine-Derived Fungal Strain *Talaromyces albobiverticillius* 30548

**DOI:** 10.3390/microorganisms7010010

**Published:** 2019-01-08

**Authors:** Mekala Venkatachalam, Léa Gérard, Cathie Milhau, Francesco Vinale, Laurent Dufossé, Mireille Fouillaud

**Affiliations:** 1 Laboratoire de Chimie des Substances Naturelles et des Sciences des Aliments—LCSNSA EA 2212, Université de la Réunion, 15 Avenue René Cassin, CS 92003, F-97744 Saint-Denis CEDEX 9, Ile de la Réunion, France; mekalavenkat@gmail.com; 2 Ecole Supérieure d’Ingénieurs Réunion Océan Indien—ESIROI Agroalimentaire, 2 Rue Joseph Wetzell, F-97490 Sainte-Clotilde, Ile de la Réunion, France; lea.gerard1@agroparistech.fr (L.G.); cathie.milhau@univ-reunion.fr (C.M.); 3 Institut des Sciences et Industries du Vivant et de L’Environnement du Centre Paris Claude Bernard, 16, rue Claude Bernard, F-75231 Paris CEDEX 05, France; 4 Istituto per la Protezione Sostenibile delle Piante (IPSP-CNR/Dipartimento di Agraria, Università degli Studi di Napoli Federico II, IT-80055 Portici (NA), Italy; francesco.vinale@ipsp.cnr.it

**Keywords:** sea salts, *Talaromyces albobiverticillius*, Réunion Island, fungal pigments, biomass, color coordinates

## Abstract

Marine-derived fungi that inhabit severe changing environments have gained increasing interest for their ability to produce structurally unique natural products. Fungi belonging to the *Talaromyces* and the close *Penicillium* genera are among the most promising microbes for bioactive compound production, including colored metabolites. Coupling pigment producing capability with bioactive effectiveness would be a valuable challenge in some specific fields such as dyeing, cosmeceutical, or food industries. In this sense, *Talaromyces albobiverticillius* 30548, a red pigment producing strain, has been isolated from the marine environment of Reunion Island, Indian Ocean. In this research, we analyzed the effect of temperatures (21–27 °C) and salinity levels (0–9%) on fungal growth and pigment production. Maximum pigment yield was obtained in non-salted media, when cultured at 27 °C after 10 days of submerged fermentation in PDB. However, maximum dry biomass production was achieved at stressed condition with 9% sea salts concentrated media at the same temperature. The results indicate that salinity of the culture media positively influences the growth of the biomass. Inversely, pigment production decreases with increase in salinity over 6%. Color coordinates of secreted pigments were expressed in CIELAB color system. The hue angles (h°) ranged from red to yellow colors. This indicated that the color distribution of fungal pigments depends on the salinity in the culture media. This study emphasizes the impact of abiotic stress (salt and temperature) on the growth and metabolome of marine-derived fungal strains.

## 1. Introduction

In the search for new active molecules, filamentous fungi stand out for producing a diverse range of bioactive compounds of interest with positive or negative effects on human health (antibiotics, enzymes, organic acids, toxins, and pigments) [1,2,3]. Several novel compounds with bioactive properties have been already isolated from strains/ species in the genera *Aspergillus* [4,5], *Penicillium* and *Talaromyces* [6]. Considering pigments, divergent sources of filamentous fungi produce different chemical classes of pigments such as carotenoids, melanins, flavins, phenazines, quinones, and more specific monascins, violacein, and indigo [7,8,9,10]. These compounds have potential interest in the agrochemical, biotechnological, food, and pharmaceutical industries [11,12]. Moreover, the use of natural colorants is now gaining interest due to the increasing demand for natural and healthy products. Marine-derived fungi are considered as a prolific source for producing pigments with unique structure and bioactivities. This has increased the attention of natural products chemists and researchers to scrutinize the diversity, chemistry, and biology of marine fungi [13,14,15].

Many colored extrolites produced by filamentous fungi are polyketides, and several papers report that this class of secondary metabolites seems to dominate marine natural products of fungal origin [13,16,17,18]. Opposite to the primary metabolism, converging to few products common to many organisms, the secondary metabolic pathways diverge to a great diversity of molecules. Due to their properties, pigments are involved in several biological processes such as protection, interactions with other organisms (competition, symbiosis), metal transport, differentiation, etc. [19]. The biological significance of polyketide pigments involves resistance to a variety of adverse environmental factors (desiccation, exposure to extreme temperatures, irradiations), to antimicrobial activity. Many observations have also been published with regard to the antioxidant activity of pigments’ protective action against lethal photo oxidation, inhibition of mutagenesis, enhancement of the immune response, and inhibition of tumour development [19,20,21,22,23,24,25].

Pigment biosynthesis is influenced and regulated by a complex set of factors including temperature, pH, osmolarity, di-oxygen availability, light exposure, nature and abundance of nutritive sources, as well as age and specialization of the fungal structures [26,27,28,29,30]. However, while the underlying mechanisms of secondary metabolite’s biosynthesis gene clusters regulation are being progressively unveiled, the factors and pathway involved are poorly understood until now [31,32]. 

Increasing evidence has proved that, under culture conditions mimicking marine environmental factors, particularly in the presence of sea salts, unique secondary metabolites have been found to be produced as compared to the related strains of terrestrial/fresh water origin [33,34,35]. Novel classes of secondary metabolites were then identified from the salt-water cultures of *Penicillium chrysogenum* [34], *Aspergillus terreus* [36], *Aspergillus niger* [37], or *Trichoderma harzianum* [38]. 

Therefore, samples from different habitats of marine environment around Reunion Island, located 800 km east of Madagascar (Indian Ocean), have been collected [39]. Several strains producing pigments have been isolated. Among the fungal isolates, a strain of *Talaromyces* (*T. albobiverticillius* 30548) was found to originally synthesize a large amount of pigments in red orange hues with interesting coloring properties [40]. In a preliminary study, 12 different compounds were detected from intracellular and extracellular extracts of fungal liquid cultures, and four were tentatively identified as *Monascus*-type pigments. Three of them are similar to the already known N-threonine-monascorubramine, N-glutaryl-rubropunctamine and PP-O, respectively. Furthermore, one compound revealed a new structure named as 6-[(*Z*)-2-carboxyvinyl]-N-GABA-PPV (or N-GABA-PPV-derivative) [41].

So far, only a few published research articles deal with the effects of salts (mostly NaCl) on growth, extrolites, and pigment production by marine-derived filamentous fungi [31,32,42,43]. Nevertheless, it is widely accepted that small variations in the culture conditions can strongly impact the behavior and the production of microbes [28,44,45]. This can be a major issue in industrial fields or, oppositely, a notable asset for the synthesis of specific compounds. This work was designed to investigate the tolerance to specific stress conditions through the effects of temperature and salinity on the marine-derived *T. albobiverticillius* 30548. Thus, the fungal growth and the intensity of pigment secretion were investigated under submerged fermentation in culture medium supplemented with artificial sea salts.

## 2. Materials and Methods 

### 2.1. Microorganism and Preparation of Seed Media 

The studied fungus was isolated from sediment source in the external slope of the site Trou d’Eau (22°5′23.99′′ S, 55°14′7.03′′ E) around Reunion Island (Indian Ocean). This fungal isolate is identified as *Talaromyces albobiverticillius* through genomic sequencing using T10/Bt2b and V9G/LS266 primer pairs targeting β-tubulin and ITS regions respectively (Westerdijk Fungal Biodiversity Institute, The Netherlands). The fungus was stored in the laboratory collection of LCSNSA (Laboratoire de Chimie des Substances Naturelles et des Sciences des Aliments, Université de La Réunion) under the reference code 30548 at −80 °C for long-term storage. The culture was also maintained on PDA slants (Ref. 254920, BD Difco, USA supplemented with agar 20 g/L) at 4 °C, and sub-cultured at regular intervals. 

Seed media was prepared by taking 80 mg of mycelia from Petri plates of seven-day old culture grown on PDA at 24 °C in a sterile Eppendorf tube and vortexed with 1 mL of distilled water. Then, it was transferred into 250 mL Erlenmeyer flask containing a volume of 80 mL sterile potato dextrose broth (PDB, Ref. 254920, BD Difco, Franklin Lakes, NJ, USA) under sterile conditions and incubated at 24 °C, under 200 rpm agitation for two days (Multitron Pro, Infors HT, Bottmingen, Switzerland).

### 2.2. Preparation of Culture Media and Submerged Fermentation

To study the relationship between the temperature and fungal growth as well as pigment production, flask culture experiments were initially performed at three different temperatures (21, 24, and 27 °C) using sterile PDB without the addition of sea salts (0%). For inoculum preparation, 20 mL of homogenous two-day old liquid pre-culture was allowed to centrifuge at 8000 rpm for 6 min (Centrifuge 3K 3OH, SIGMA- Aldrich, St. Louis, MO, USA) at room temperature. After centrifugation, the supernatant was discarded and 80 mg of pellet (filamentous mycelia) settled at the bottom of the tube was taken and added to 1 mL sterile distilled water in sterile Eppendorf tubes, then vortexed. Furthermore, the vortexed content was inoculated to each flask under sterile conditions and incubated at 21, 24, and 27 °C for 10 days with an agitation speed of 200 rpm. After temperature optimization, fermentation using different sea salt concentrations was performed at 27 °C.

The culture media was then prepared using PDB supplemented with artificial sea salts (Sigma Sea Salts S-9883). The percentage of salinity was varied at four different levels (0, 3.65, 6, and 9%). A volume of 80 mL media was dispensed into 250 mL Erlenmeyer flasks. The initial pH was adjusted to 4.0 using 2M HCl solution prior to sterilisation in the autoclave, and the flasks were autoclaved at 121 °C for 15 min and then left to cool at room temperature. All experiments were performed in technical triplicates which originated from the same inoculum in a one-shot experiment (not biological triplicates that would be produced by three different seed inoculum) following the above-mentioned conditions of inoculum preparation and fermentation. 

### 2.3. Monitoring Methods 

To monitor the fungal growth and pigment production throughout the entire fermentation period, 5 mL of fermented broth from each flask were sampled once every 24 h. The pH was measured and recorded for each flask using a pH meter (pH 1500, Eutech Instruments, with a Bioblock Scientific probe, Thermo Fisher Scientific Inc., Waltham, MA, USA). The samples were then filtered using labeled nylon cloth of pore size 48 µm (Nitex, SEFAR AG, Heiden, Switzerland) to separate the biomass and supernatant.

### 2.4. Determination of Dry Biomass 

The amount of wet biomass obtained after filtration was noted using a precise analytical weighing balance (Adventurer Pro AS214 d = 0.0001 g, Ohaus Europe GmbH, Greifensee, Switzerland). To determine the dry biomass weight, the wet filters were dried in a hot air oven (SNB 100, Memmert, Schwabach, Germany) at 105 °C for 17 h. The filters were weighed after keeping in desiccator for 30 min to bring it at room temperature [46].

### 2.5. Estimation of Pigment Absorbance and Standard Curve 

Absorbance of pigments in the extracellular culture filtrate was measured using UV-1800 Spectrophotometer (Shimadzu, Kyoto, Japan). The colored culture filtrates were scanned at 230–700 nm for maximum wavelength absorbance of the pigments. Absorbance of extracellular pigments from fungal liquid culture was read at 500 nm, which represent a widespread maximum for orange and red pigments respectively [47]. The pigment yield was expressed in terms of g/L *Monascus* red rice equivalents. This was done by extrapolating the absorbance vs. concentration (g/L) calibration curve of commercially available red yeast rice (Wuhan Jiacheng Biotechnology Co., Ltd., Wuhan, China).

*Monascus* red rice is a product obtained by fermentation using *Monascus purpureus* and considered as one of the food supplements by having active main constituents [48]. It was used as a reference standard, since the pigments produced by *T. albobiverticillius* 30548 were found to be *Monascus*-like pigments, reported by HPLC-MS and NMR studies.

The concentration of the pigments was calculated using the following formula

(1) $$C=\frac{Abs_{500}-0.0097}{1.6925}$$

where,

C = concentration of pigments in g/L
Abs_500_ = absorbance at 500 nm wavelength

The gradient (0.0097) and intercept (1.6925) represent the values obtained from the standard curve of *Monascus* red rice. 

### 2.6. Color Analysis of Pigments

The extracts used for absorption scanning were again used to determine CIELAB color coordinates. The color measurements were performed using Spectrocolorimeter (CM-3500d Spectrocolorimeter, Konica Minolta, Japan) and the values of L*, a*, b*, *C*, and *h*° were obtained automatically with the help of SpectraMagic™NX which is a color data software (version 1.9, Minolta Co., Tokyo, Japan). Color measurements were performed on alternate days with a 30 mm filter (Konica Minolta, Tokyo, Japan). The standard illuminant D65 was used throughout the colorimetric measurements intended to represent average daylight. The colorimetric system defines the characters, L for brightness ranging from 0 (black) to 100 (white), where a* represents the change from green (negative values) to red (positive values); b* represents the change from blue (negative values) to yellow (positive values). Chroma is the color strength of the object, denoted by *C* and hue is the saturation or purity of a color, represented as *h*°. Values close to the centre at the same L* values express dull or grey color, though values near circumference indicates bright or vivid colors [49].

Chroma and hue angle are calculated from a* and b* coordinates in L*a*b*.

(2) $$Chroma\cdot C^{*}=\sqrt{{\left(a^{*}\right)}^{2}+{\left(b^{*}\right)}^{2}}$$

(3) $$Hue\cdot angle\cdot h^{{}^{\circ}}=tan^{-1}\left(\frac{b^{*}}{a^{*}}\right)$$

### 2.7. Quality Control

Random samples were taken from several flasks during the early fermentation period to examine the phenotypic characters under a light microscope as well as to check the purity of the culture (CX41, Olympus, Tokyo, Japan).

### 2.8. Statistical Analysis 

All the experiments were performed in technical triplicates to calculate the means and standard deviations. The statistical functions and the corresponding descriptive graphs were created using Sigmaplot software version 11 (Systat Software Inc., San Jose, CA, USA). To determine the adequacy of fit data and its significance, one-way analysis of variance (ANOVA) was performed to compare the mean values of individual variable between the four conditions followed throughout the experiment at 95% significance level. For all the treatments, the exponential growth phase was modelled through a linear model and the slope was calculated to determine the fungal growth rate.

## 3. Results

### 3.1. Influence of Temperature on Fungal Growth and Biomass

**Figure 1:**
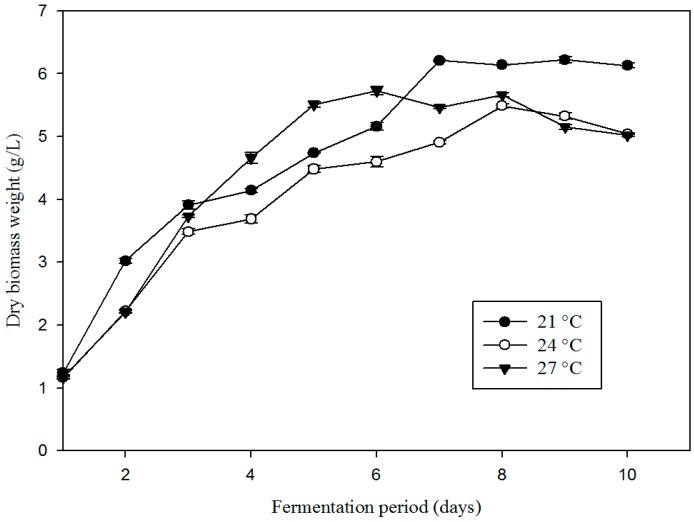
Compared effects of culture at three different temperatures (21, 24, and 27 °C) on biomass growth for *T. albobiverticillius* 30548 (culture in PDB, initial pH 4.0, 200 rpm of agitation rate).

For a better understanding of temperature influence on fungal growth rate and biomass production, fermentation was carried out at three different temperatures (21, 24, and 27 °C) (Figure 1).

With dry weight of fungal biomass used as a criterion, the biomass production rates were calculated during the maximal growth phases, depending on the experimental conditions (1 to 7 days for 21 °C, 1 to 8 days for 24 °C, and 1 to 5 days for 27 °C). The weight of final dry biomass was set once the growth had stopped completely in the stationary phase (day 10 for all cultures) (Table 1).

Growth rate was significantly higher at 27 °C (1.11 g/L/day) in comparison to 21 and 24 °C (*p* = 0.0009 < 0.05), both leading to similar growth rates. The final dry biomass weights ranged from 5.48 ± 0.07 to 6.22 ± 0.12 g/L. These results significantly showed that the fungus cultured at the temperature of 27 °C grew faster during the first five days of exponential growth. However, the final biomass produced after 10 days was significantly higher at 21 °C, compared to the growth at higher temperatures (*p* = 0.0004 < 0.05). This implied that the temperature and time factor had a significant influence on fungal growth and behavior (length of the exponential growth phase) in flask cultures.

### 3.2. Influence of Temperature on Pigment Production

**Figure 2:**
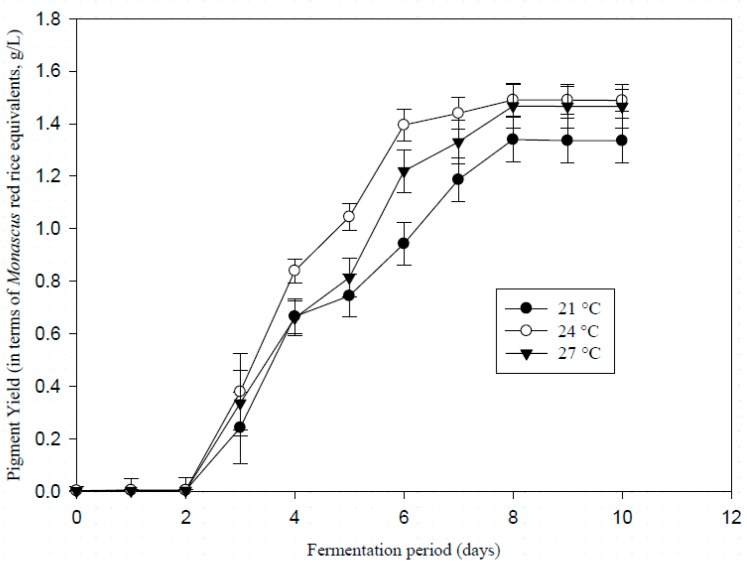
Compared effects of culture at three different temperatures (21, 24, and 27 °C) on red pigment yield (expressed in *Monascus* red rice equivalents g/L, measured at 500 nm) for *T. albobiverticillius* 30548 (culture in PDB, initial pH 4.0, 200 rpm of agitation rate).

The fungal strain produces red pigments at the chosen three different temperatures. In all the treatments (21, 24, and 27 °C) pigment production was initiated on day 3 and a large deviation among the technical triplicates was seen in the beginning of exponential phase, which is clearly indicated in Figure 2. This demonstrates the asynchronous nature of the technical triplicates in terms of pigment production, when starting a new culture. Also, a significant delay takes place between the beginning of the biomass increase (day 1) and the beginning of the pigment production (day 3). This shows the true nature of pigmented molecules as secondary metabolites. The maximum pigment production was attained on day 8 for 21 and 27 °C then followed by the stationary phase. However, at 24 °C, pigment production appeared a bit more efficient because it nearly reached the maximum on day 6, and the overall curve is located above the two others since from the beginning. 

In addition, rates of pigment production during the exponential growth of the fungi as well as the final pigment yields were calculated, in terms of *Monascus* red rice equivalents (Table 2). 

Results from one-way ANOVA indicate that there was no significant difference between the means obtained at the three temperatures (*p* = 0.139 > 0.05 for production rates and *p* = 0.145 > 0.05 for maximum pigment yield). The maximum values obtained were 0.36 g/L/day as growth rate and 1.49 ± 0.06 g/L for pigment yield in terms of *Monascus* red rice equivalents.

Hence, the temperatures in the chosen range did not exert a strong influence on the production of pigments in *T. albobiverticillius*, while it significantly influenced the biomass growth. 

### 3.3. Influence of Sea Salts on Fungal Growth Rate and Dry Biomass Weight

**Figure 3:**
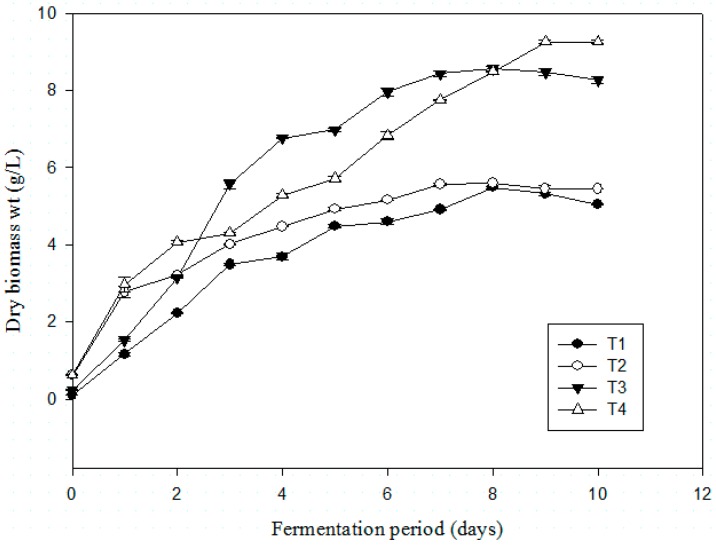
Effect of different levels of sea salts concentration (0% (T1), 3.65% (T2), 6% (T3), 9% (T4)) on dry biomass weight for *T. albobiverticillius* 30548 (culture in PDB, 27 °C, initial pH 4.0, 200 rpm of agitation rate).

The behavior of the strain towards different salt treatments was tested in PDB supplemented with 0% (T1), 3.65% (T2), 6% (T3), and 9% (T4) (w/v) sea salts (Figure 3). Dealing with fungal behavior, no lag phase was observed before the beginning of growth. This could be explained by the fact that the inoculum adapted very well to all the different conditions, thus the growth could start immediately.

The significance of the differences between the fungal growth rates during exponential phase as well as between the final biomass contents was statistically analysed using ANOVA (*p* ≤ 0.05) and Tukey’s test (Table 3). Significant differences were detected between 0% (or) 3.65% and 6% and also 9% (*p* = 0.005 < 0.05) as well as between 6% and 9% (*p* = 0.005 < 0.05). 

From the plotted results, it can be observed that T4 exhibited linear increase from day 1 until maximum biomass yield obtained on day 9. T2 and T3 obtained maximum biomass yield earlier on day 7. Then, there is a clear difference in the final dry biomass weights obtained in salted media, which was higher for T3 and T4. From Table 3, it is also highlighted that the final dry biomass were not significantly different from T1 or T2. Thus, the addition of sea salts was found to significantly increase the final dry biomass weight in *T. albobiverticillius* 30548 from 6% and above (up to 9.22 ± 0.09 g/L), and it also had a positive impact on the growth rates.

### 3.4. Influence of Sea Salts on Pigment Production 

**Figure 4:**
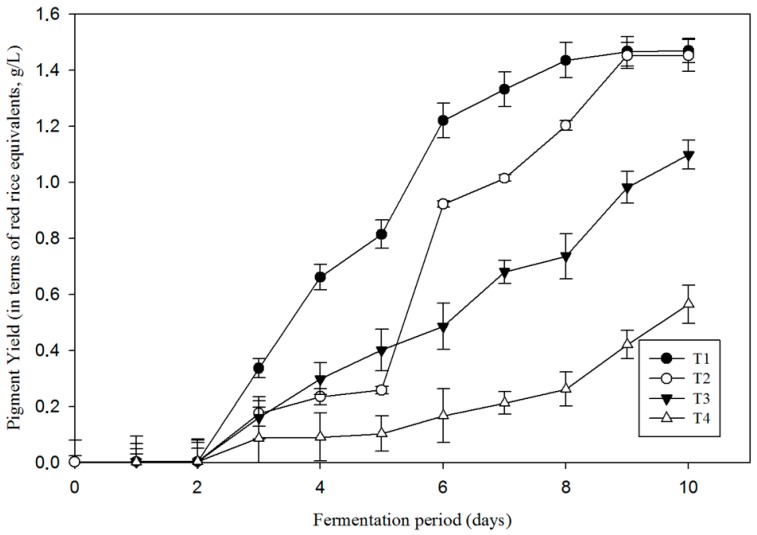
Effect of salt concentration (0% (T1), 3.65% (T2), 6% (T3), 9% (T4)) on pigment yield in *T. albobiverticillius* 30548 (expressed in *Monascus* red rice equivalents g/L, measured at 500 nm) (culture in PDB, 27 °C, initial pH 4.0, 200 rpm of agitation rate).

The rates of pigment production for different levels of salinity are presented in Figure 4. Enhanced pigment production was noticed in T1 then followed by T2. The time required for initiation of visible pigment production was also found to be higher in saline media when compared to 0% salt concentration. From the results, it was noticed that T1 exhibited a prominent pigment production rate from day 2 to day 7 (Table 4). T2 exhibited a short delay in pigment production until day 5, then it showed a drastic increase until day 9, when it reaches the maximum pigment yield. 

To test the statistical significance between the pigment production rates, *t*-testing was performed between the treatments. The difference between T1 and T2 was not significant (*p* = 0.055> 0.05) while a significant difference exists among the treatments T1, T3, and T4, which is given by the *p*-value 0.002 < 0.05. The highest production rate observed for T1 (0.27 g/L/day in terms of *Monascus* red rice equivalents).

The final pigment yields were similar for treatment T1 and T2 (with a maximum value 1.46 ± 0.08 g/L). For treatment T3 and T4, the final pigment yields were significantly lower compared to the other media. 

Salt contents in the medium thus had a significant influence on the pigment yield and its production rate (*p* = 0.002 < 0.05). This influence is also visible through changes in color values.

**Figure 5:**
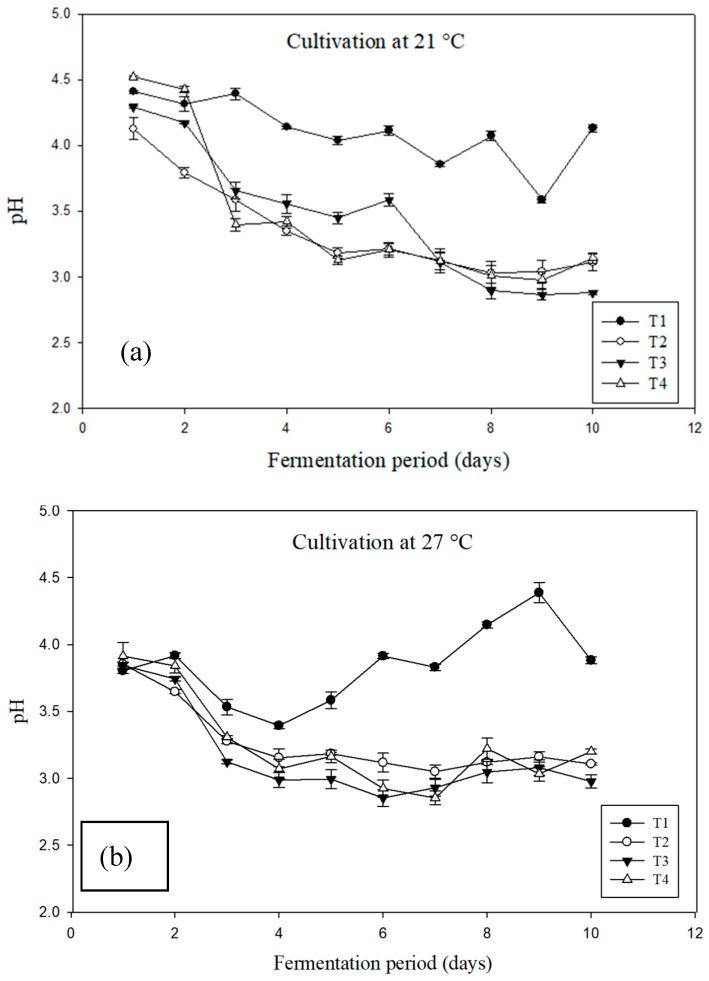
pH evolution of *T. albobiverticillius* strain 30548 liquid cultures grown at (**a**) 21 °C and (**b**) 27 °C with increasing concentration of sea salts (T1 0%, T2 3.65%, T3 6%, T4 9%)

Concurrently, pH of the culture medium plays a key role in pigment synthesis. Based on the experimental observations, it was noted that in the presence of salt, the fungal cultures strongly decrease the pH of the medium, at all concentrations. Whereas for the control media without the addition of sea salts (T1), pH decreased to a smaller extent at 21°C or even increased after day 5 at 27 °C, both cultures attaining higher red pigment production (Figure 5). In order to avoid such pH variations, the next cultures investigating salinity effect on pigment production by *T. albobiverticillius* will be conducted in a 10-liter fermenter in a pH-regulated mode (pH stable value at 4.0 during all the fermentation).

### 3.5. Effect of Sea Salts on Color Hues

The commercially available Monascus red rice (R) and orange quinizarin (O) were used as reference colorants (1 g/L) to compare L a* b*C h° color values with fungal culture filtrates from *T. albobiverticillius* 30548. The a* values of all the cultured filtrates were found to be positive ranging from 6.87 to 81.25 (pale pink to red) and similarly for b*, only positive values were measured, from 21.25 to 46.08 (grey to yellow) (Table 5). 

**Figure 6:**
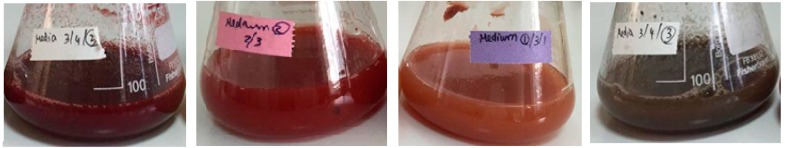
Observation of the different shades of pigments produced in different saline media (from left to right, 0, 3.65, 6, 9%).

The hue angles (*h*°) of the culture filtrates (0, 3.65, and 6% w/v sea salts) ranged from 23.07 to 37.08 signifying red-orange color, while, the hue angle of 9% sea salts concentration is very close to yellow region (Figure 6). In the culture media with low percentage of sea salts (0% and 3.65%), “dark red” hue was observed. Instead, salts concentrated media (6%, 9%) altered the color from pale orange to grey/brown indicating a clear change in its pigment content and structure in terms of orange and red components (Figure 5). This implies that addition of sea salts to the culture medium modified the color of the pigment mixture produced by *T. albobiverticillius* 30548. To further explain and clarify, new pigment structures should be present in cultures with sea salts, as the azaphilone pigments already described in *T. albobiverticillius* 30548 are not pH indicators such as anthocyans from plants; the azaphilone hues remain stable in a very large range of pH.

**Figure 7:**
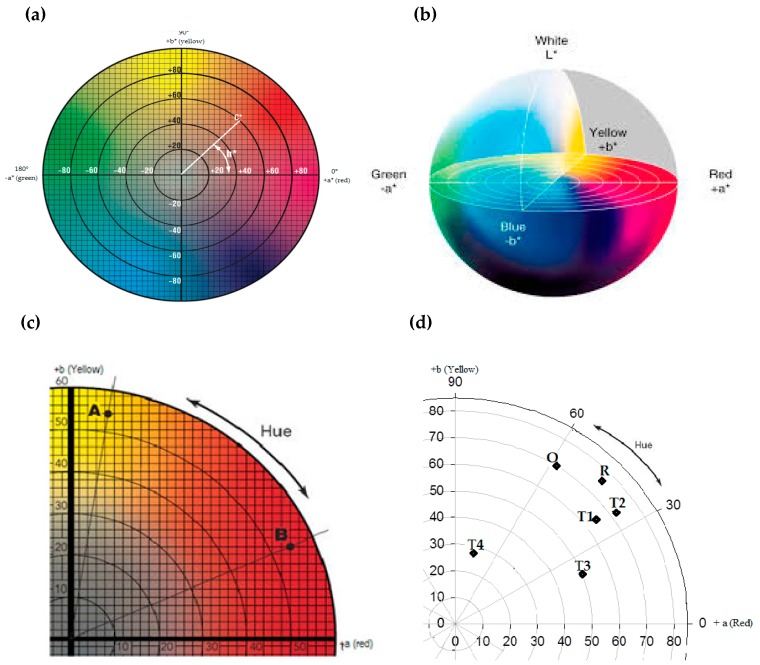
(**a**) CIE L*a*b* chromaticity diagram (Konica Minolta precise color communication). (**b**) Two dimensional L*a*b* model from CIE L*a*b* color space. (**c**) Representation of CIE L*a*b* colorimetric system showing the hue angles (*h*°). (**d**) Polar scatter plot showing the positions of pigments of *T. albobiverticillius* 30548 cultured in four different sea salt concentrated media: T1 (0%), T2 (3.65%), T3 (6%), T4 (9%) including reference standards, quinizarin (O) and *Monascus* red rice (R).

T1, T2, and T3 exhibit close hue angles compared to T4, which appears completely different (Figure 7). Moreover, the hue angles of T1 and T2 seem closer to the reference colorant, Monascus red rice (R). Whereas the hue angle of T4 is more in the angle of quinizarin (O), the orange reference, with a lower lightness. The saturation, C* responses of the four different treatments (T1, T2, T3, T4) widely ranged from 30.62 to 76.14. Next to the treatment T2, T1 exhibited significantly higher concentration of pigments, which is represented by high saturation values (76.14 and 71.75 respectively). Comparatively, T3 and then T4 are positioned lower (54.20, 30.62). The saturation of the color is also clearly modified by the variations in salt concentrations in the culture media. 

## 4. Discussion

### 4.1. Temperature Effects

Numerous studies on various fungal species have reported that temperature has a great impact on cell development [50]. The range 21–27 °C was chosen to design the in vitro growth curve of *T. albobiverticillius* 30548, isolated from a tropical marine area at −17 m. In this study, incubation at the lowest temperature (21 °C) significantly increased biomass final level, but did not have significant effect neither on pigment production rate, nor on the final amount of colored metabolites. Thus, in flasks, this tropical fungus grows faster at 27 °C and the pigment production is not affected by variation of temperature in this range.

Several studies reported that changes in temperature (either increase or decrease) induce changes in the production of pigments. In this regard, Babitha et al. [51] described that increasing the temperature to 30 °C resulted in higher red pigment production in *Monascus purpureus* LPB 97 at 500 nm, while increment of temperature to 40 °C produced more yellow pigments absorbing at 400 nm. 

### 4.2. Salinity Effects

Since our interest was to test the tolerance of marine-derived fungi to high salt stress and the reaction towards pigments production, we have used artificial sea salts to study *T. albobiverticillius* growth and production in liquid media. From Aujla [52], the water potential of PDB can be evaluated around −0.32 MPas (1 Mega Pascal = 10 bars) at 25 °C. Sea-salts mix from Difco mimics the composition of salt mixture in sea water, whose salinity is approximately 36.5 g/L in tropical regions. As NaCl is the major component of sea-water salts it is possible to roughly assess the water potential of PDB supplemented with 3.65% sea salts at −3 MPas (−5 MPas at −6% and −8 MPa at 9%). 

The present study shows a remarkable influence of sea salts on the biomass growth of *T. albobiverticillius* 30548. Under high saline conditions (6% and 9%) in the culture media, corresponding to approximately −5 and −8 MPas, this marine-derived fungus exhibited faster and higher biomass development, indicating this species is particularly well adapted to salt stress. This was in agreement with the work done by Masuma et al. [53]. 

In *Aspergillus glaucus*, salt stress highly enhanced primary metabolism covering increased biomass growth, but showed little influence on secondary metabolism in terms of pigment production with regards to quantity [54]. Indeed, fungal pigments are usually produced after the active growth of the organism. They are bound to appear when the environmental conditions become unfavourable, in particular when a substrate other than carbon becomes limited [55]. In this work, the pigments effectively started to be produced in the middle of the logarithmic growth phase (on day 3) and reached maximum at the end of the exponential phase, which is a usual phenomenon for secondary metabolites. However, in *T. albobiverticillius* 30548, the pigment production was significantly less efficient when the salinity increased.

Tolerance to high salt stress is a property of marine-derived fungi, predominantly some species of *Aspergillus* and *Penicillium* [56]. Huang et al. [43] studied the effects of salinity on growth, secondary metabolites production and biological activities of several marine-derived strains of *Penicillium*. In their report, it was found that NaCl effectively promoted growth in 91.5% of their 47 strains, and antimicrobial activity in 14.5%. These results confirm that salinity is a crucial factor influencing growth and secondary metabolites production [33]. In 1957 and 1959, Ritchie also found that the growth of various tropical marine-derived fungi can be faster at higher salinities while maintaining at high temperatures [57,58]. Extending this evidence to some strains, a similar pattern was observed in *Aspergillus nidulans*, *Curvularia* sp., *Dendryphiella salina*, *Penicillium chrysogenum*, *P. citrinum*, *P. corylophilum P. dravuni*, *Pestalotia* sp., *Phoma* sp. [59,60,61,62]. However, most fungi were found to be more halotolerant than halophilic and do not require salt concentrations for their viability [63]. Obviously, the microbial growth in highly saline environments requires numerous adaptations more specifically at cell wall level by maintaining osmotic homeostasis of cells [64,65,66,67]. Thus, many studies revealed that fungi cultured in highly saline medium undergo decreased growth when compared to control. The salinity tolerance level in terms of NaCl concentrations towards growth was then discussed for some *Aspergillus* spp. (4%), *Cladosporium* (4%), *Pestalotiopsis* (4%) [53]; *Penicillium* sp. (3–6%) [43]; *Microporus xanthopus* (3.5%), *Pycnoporus sanguineus* (6%), and *Schizophyllum commune* (7%) [68]. Furthermore, growth was suppressed if the concentration increases above the mentioned tolerance level.

Oppositely to biomass increase, the production of pigments in *T. albobiverticillius* was significantly less efficient with increasing salts concentrations during the 10 days of culture. Moreover, the time required for initiation of visible pigment production in saline media was found to be higher when compared to 0% salt concentration. This could be explained by an initial “adaptation-time” which might have been required for the fungus in saline media, as the seed culture was grown in PDB without sea salts [69]. However, present findings were in agreement with Chintapenta et al. [70], who noticed a decreased production of red pigments in *Penicillium* sp. when the salt concentration was increased to 2% (w/v) in the culture media. First, it is possible that the oxygen limitation may be related to the low pigment production observed. Indeed, literature reports that viscosity in the fermentation medium progressively increases from the initial negligible values, due to the increasing cells concentration and the release of any metabolites into the medium [71,72]. High viscosity of the media may result in reduced oxygen transfer and increase fraction of oxygen limited-cells. Ahn et al. [73] observed a similar pattern of decreased *Monascus* pigment production, linked to the increased viscosity which was due to the highly entangled mycelial clumps. Additionally, it was reported that most of the pigments adhered to the mycelia instead of dissolving into the media with increased salt concentration (from 8 to 10% NaCl) [70]. In our study, there was diffusion of red pigments but the concentration of secreted pigments was lower, may be partly due to the change in pH level (Figure 5) and alteration of osmolarity. The change in pH may induce the production of different molecules. Also, it could be explained that the increasing salt concentration altering the pH and osmolarity of the media may prevent the diffusion of pigments, or modify the metabolism. Indeed, pigments were widely diffused into the medium without additional salts; hence the water-soluble pigments gave a dark red hue compared to highly saline medium [74]. Further study of this strain exposed to different pH ranges coupled with saline medium as well as evaluation of intracellular pigments content may provide an insight in the fungal physiological behavior. 

On the contrary, Babitha et al. [51] proposed significantly larger amount of red pigments production in high salinity at 10% NaCl for *Monascus purpureus* LPB97 cultured on solid state fermentation for 12 days period. Besides, in a strain of *Talaromyces verruculosus*, the pigment production was hampered due to stress induced by NaCl [75]. 

### 4.3. Differences in Color Hues

Color diversity of extracellular pigments in the culture filtrates of *T. albobiverticillius* 30548 was analysed using spectrocolorimetric system. Alignments of hue values ranged from 23.07 to 77.04 exhibited the shades from pink to yellow depending on salt levels in the medium. Similarly, for *P. chrysogenum* IFL_2_ hue angles of all of the fungal extracts ranged from 1.57 to 90.41 when cultured in PDB [76]. Based on these differences, the color varied from pink to red to orange and from yellow to light green-yellow. Moreover, literature already mentions that in the presence of marine salts, fungi show different metabolic profiles [13], undoubtedly impacting the pigment production. To our knowledge, this is the first report to deal with the color characteristics of red pigments produced from marine-derived *T. albobiverticillius* cultured in salt enriched medium.

**Figure 8:**
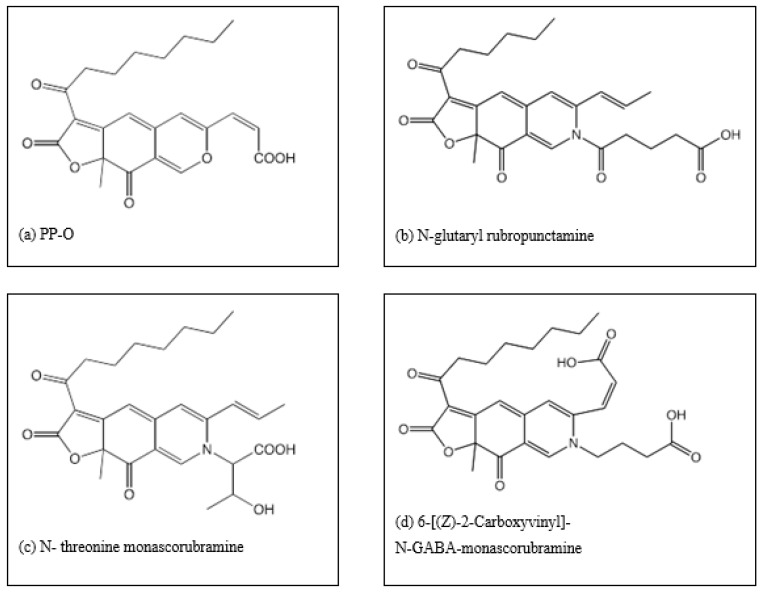
(**a**–**d**) Chemical structures identified in *T. albobiverticillius* 30548 cultured at 24 °C in potato dextrose broth without the addition of sea salts. (**a**) PP-O, (**b**) N-glutaryl rubropunctamine, (**c**) N- threonine monascorubramine, (**d**) 6-[(Z)-2-Carboxyvinyl]-N-GABA-monascorubramine (from reference [41]).

Investigation of colored extracts biosynthesized in each salinity or temperature experimental condition is planned using HPLC coupled with DAD and MS. The preliminary chemical data clearly demonstrate that pigment production in *T. albobiverticillius* 30548 is basically a mixture of several compounds [41]. Studies on HPLC characterization detected 12 different compounds from *T. albobiverticillius* 30548 cultivated in the media without adding sea salts. Among the 12 compounds, 4 were tentatively identified as *Monascus*-type pigments and the chemical structures are represented in Figure 8a–d. A new compound, named 6-[(Z)-2-Carboxyvinyl]-N-GABA-PP-V, (or as N-GABA-PP-V) was determined from PDA, MS and NMR spectral analyses (Venkatachalam et al. [41]). Among them, some compounds are produced in major quantities while some others are in lower amounts. The molecules characterized in the defined set of conditions belong to azaphilones. This class is well known to exert a large range of bioactivities [77,78,79,80]. 

Martinkova et al. [81] reported that the orange pigments, monascorubramine and rubropunctamine exerted antibiotic activity, depending on the composition of culture media and method of culture. Also, these compounds had been shown to have embryo toxicity and teratogenicity. Some of the compounds are of *Monascus*-type, and at least one is a new derivative of PP-V. As the color is clearly changing when the culture conditions are modified (in terms of salinity), the proportions and the nature of the pigments produced may also vary. This fungal isolate *T. albobiverticillius* 30548 is furthermore interesting as several strains of *T. albobiverticillius* already studied by us and other researchers did not produce any toxin. Thus, this strain can be considered as a promising source for industrial production of red pigments [29].

## 5. Conclusions

The marine-derived strain *T. albobiverticillius* 30548 subjected to various sea-salt levels (0–9%) was studied in order to understand the impact of salinity on fungal growth and pigment production. Through this investigation, the isolated marine-derived fungus appeared to grow faster at 27 °C but to spread more efficiently at 21 °C. It also demonstrated its halophilous nature by producing higher biomass yield at high salinity. Indeed, *T. albobiverticillius* presented a quick adaptation to salinity in broth medium with 6% sea salts (T3) with higher biomass production compared to T1 (0%) and T2 (3.65%). Moreover, treatment T4 (9%) roughly equivalent to −8 MPas, gave the maximum dry biomass weight (9.22 ± 0.09 g/L). Oppositely, the pigment production was not effective at higher percentage of saline media, since the highest level was achieved in 0% sea salts medium. Therefore, as the production of pigment is often described as a protective action against a variety of stress factors, we may hypothesize that in the conditions of our experiments, high red pigment production from *T. albobiverticillius* 30548 grown in low salinity media (0% and 3.65%) is a reaction toward stress. This protective reaction is decreased when salinity increases (6% and 9%) showing that this isolate is particularly well adapted to low water potential environments (−8 MPas).

The major colored compounds obtained from the marine-derived strains of *T. albobiverticillius 30548* have been characterized as azaphilones. In this regard, much work is needed to identify the exact composition and the bioactivity of the pigmented compounds produced by this strain. Some modifications in the parent compounds may also appear when the percentage of salinity varies in the culture media. This should be investigated using liquid chromatography coupled with time-of-flight mass spectrometry. Further studies on pigment isolation and characterization are needed in order to fully exploit the variation in pigment production by *T. albobiverticillius* to varying salinity concentrations. In this current era towards seeking for natural pigments from microbial sources, extremophilic behavior of halophilic fungi makes them particularly interesting candidates for biotechnological applications. The modifications of the pigments’ nature in relation to the modification of the culture media may pave the way of guided biosynthesis. Indeed, they may demonstrate their capabilities to produce different compounds by resting on its particular physical conditions. Certainly, the total findings may be useful for designing selective experiments to further understand the pathways associated with differences in metabolite formation with salt stress.

## Figures and Tables

**Table 1:** Biomass growth rates and final dry biomass weights based on temperature of growth for *T. albobiverticillius* 30548 (culture in PDB, initial pH 4.0, 200 rpm of agitation rate).

Temperature	Exponential Phase	Fungal Growth Rate (g/L/day) *	SD	R^2^	Maximum Dry Biomass Weight (g/L) *	SD
21 °C	Days 1 to 7	0.71 ^a^	0.07	0.97	6.22^b^	0.12
24 °C	Days 1 to 6	0.69 ^a^	0.11	0.93	5.48 ^a^	0.07
27 °C	Days 1 to 5	1.11 ^b^	0.09	0.99	5.66 ^a^	0.09

* same letter indicates no significant difference between the values according to Tukey’s test.

**Table 2:** Pigment production rate (in terms of *Monascus* red rice equivalents) from *T. albobiverticillius* 30548 depending on temperature (culture in PDB, initial pH 4.0, 200 rpm of agitation rate).

Temperature	Exponential Phase	Production Rate (g/L/day) *	SD	R^2^	Maximum Pigment Yield (g/L) *	SD
21 °C	Days 2 to 8	0.22 ^a^	0.04	0.97	1.34 ^a^	0.08
24 °C	Days 2 to 7	0.36 ^a^	0.07	0.98	1.49 ^a^	0.06
27 °C	Days 2 to 7	0.29 ^a^	0.03	0.98	1.46 ^a^	0.08

* same letter indicates no significant difference between the values according to Tukey’s test.

**Table 3:** Rates of fungal growth and final biomass weights from *T. albobiverticillius* 30548, based on salinity in the fermentation medium (PDB supplemented with sea salts, 27 °C, initial pH 4.0, 200 rpm of agitation rate).

Sea Salts Concentration	Reference Code	Exponential Phase	Fungal Growth Rate (g/L/day) *	SD	R^2^	Final Biomass (g/L) *	SD
0%	T1	Days 1 to 6	0.58 ^a^	0.05	0.93	4.88 ^a^	0.03
3.65%	T2	Days 1 to 5	0.55 ^a^	0.09	0.96	5.01 ^a^	0.03
6%	T3	Days 1 to 6	1.28^b^	0.05	0.89	8.28 ^b^	0.06
9%	T4	Days 1 to 8	0.77^c^	0.02	0.99	9.22 ^c^	0.09

* same letter indicates no significant difference between the values according to Tukey’s test.

**Table 4:** Rate of pigment production and final pigment yield from *T. albobiverticillius* 30548 based on salinity percentage in the fermentation media (culture in PDB, 27 °C, initial pH 4.0, 200 rpm of agitation rate).

Sea Salts Concentration	Reference Code	Exponential Phase	Production Rate (g/L/day) *	SD	R^2^	Final Pigment Yield (g/L) *	SD
0%	T1	Days 2 to 7	0.27 ^a^	0.09	0.98	1.46 ^a^	0.08
3.65%	T2	Days 2 to 9	0.22 ^a^	0.07	0.93	1.45 ^a^	0.06
6%	T3	Days 2 to 9	0.13^b^	0.09	0.99	1.09^b^	0.08
9%	T4	Days 2 to 9	0.05^c^	0.08	0.90	0.56^c^	0.07

* same letter indicates no significant difference between the values according to Tukey’s test.

**Table 5:** *L*, a*, b*, *C*, and *h*° values of *T. albobiverticillius* 30548’s production on day 10 (culture in PDB supplemented with sea salts, initial pH 4.0, 200 rpm of agitation rate). *Monascus* red rice and quinizarin (1g/L) used as reference standards.

Sea Salts Concentration	Reference Code	*L*	a*	b*	*h°_ab_*	*Chroma*
0%	T1	57.12	56.73	42.94	37.08	71.75
3.65%	T2	48.16	61.38	46.08	36.89	76.14
6%	T3	75.25	49.87	21.25	23.07	54.20
9%	T4	81.25	6.87	29.87	77.04	30.62
*Monascus* red rice	R	54.02	57.41	61.97	47.19	84.48
*Quinizarin*	O	91.24	42.18	65.12	57.06	77.59

## References

[1] Hajjaj H., Blanc P., Groussac E., Uribelarrea J.-L., Goma G., Loubiere P. (2000). Kinetic analysis of red pigment and citrinin production by *Monascus ruber* as a function of organic acid accumulation. Enzyme Microb. Technol..

[2] Zhang L., An R., Wang J., Sun N., Zhang S., Hu J., Kuai J. (2005). Exploring novel bioactive compounds from marine microbes. Curr. Opin. Microbiol..

[3] Vaishnav P., Demain A.L. (2011). Unexpected applications of secondary metabolites. Biotechnol. Adv..

[4] Hasan S., Ansari M.I., Ahmad A., Mishra M. (2015). Major bioactive metabolites from marine fungi: A review. Bioinformation.

[5] Hong J.-H., Jang S., Heo Y.M., Min M., Lee H., Lee Y.M., Lee H., Kim J.-J. (2015). Investigation of marine-derived fungal diversity and their exploitable biological activities. Mar. Drugs.

[6] Nicoletti R., Trincone A. (2016). Bioactive compounds produced by strains of *Penicillium* and *Talaromyces* of marine origin. Mar. Drugs.

[7] Kirti K., Amita S., Priti S., Jyoti S. (2014). Colorful world of microbes: Carotenoids and their applications. Adv. Biol..

[8] Dufossé L., Schaechter M. (2009). Pigments microbial. Encyclopedia of Microbiology.

[9] Dufossé L. (2006). Microbial production of food grade pigments. Food Technol. Biotechol..

[10] Dufossé L., Fouillaud M., Caro Y., Mapari S.A., Sutthiwong N. (2014). Filamentous fungi are large-scale producers of pigments and colorants for the food industry. Curr. Opin. Biotechnol..

[11] Adrio J.L., Demain A.L. (2003). Fungal biotechnology. Int. Microbiol..

[12] Butler M.S. (2004). The role of natural product chemistry in drug discovery. J. Nat. Prod..

[13] Imhoff J.F. (2016). Natural products from marine fungi—Still an underrepresented resource. Mar. Drugs.

[14] Bhatnagar I., Kim S.-K. (2010). Immense essence of excellence: Marine microbial bioactive compounds. Mar. Drugs.

[15] Cragg G.M., Newman D.J. (2013). Natural products: A continuing source of novel drug leads. Biochim. Biophys. Acta.

[16] Hanson J.R. (2003). Natural Products: The Secondary Metabolites.

[17] Ebel R., Liu H.-W., Mander L. (2010). 2.08—Natural product diversity from marine fungi. Comprehensive Natural Products II.

[18] Rateb M.E., Ebel R. (2011). Secondary metabolites of fungi from marine habitats. Nat. Prod. Rep..

[19] Fouillaud M., Venkatachalam M., Girard-Valenciennes E., Caro Y., Dufossé L. (2016). Anthraquinones and derivatives from marine-derived fungi: Structural diversity and selected biological activities. Mar. Drugs.

[20] Avalos J., Carmen Limon M. (2015). Biological roles of fungal carotenoids. Curr. Genet..

[21] Berestetskiy A.O., Gasich E.L., Poluektova E.V., Nikolaeva E.V., Sokornova S.V., Khlopunova L.B. (2014). Biological activity of fungi from the phyllosphere of weeds and wild herbaceous plants. Microbiology.

[22] Dadachova E., Bryan R.A., Howell R.C., Schweitzer A.D., Aisen P., Nosanchuk J.D., Casadevall A. (2008). The radioprotective properties of fungal melanin are a function of its chemical composition, stable radical presence and spatial arrangement. Pigment Cell Melanoma Res..

[23] Gessler N., Egorova A., Belozerskaya T. (2013). Fungal anthraquinones. Appl. Biochem. Microbiol..

[24] Jančič S., Frisvad J.C., Kocev D., Gostinčar C., Džeroski S., Gunde-Cimerman N. (2016). Production of secondary metabolites in extreme environments: Food-and airborne *Wallemia spp.* Produce toxic metabolites at hypersaline conditions. PLoS ONE.

[25] Margalith P. (1992). Pigment Microbiology.

[26] Kurobane I., Vining L.C., McInnes A.G., Walter J.A. (1980). Use of ^13^C in biosynthetic studies. The labeling pattern in dihydrofusarubin enriched from [^13^C]- and [^13^C, 2H]acetate in cultures of *Fusarium solani*. Can. J. Chem..

[27] Julia P., Martinkova L., Lolinski J., Machek F. (1994). Ethanol as substrate for pigment production by the fungus *Monascus purpureus*. Enzyme Microb. Technol..

[28] Cho Y.J., Hwang H.J., Kim S.W., Song C.H., Yun J.W. (2002). Effect of carbon source and aeration rate on broth rheology and fungal morphology during red pigment production by *Paecilomyces sinclairii* in a batch bioreactor. J. Biotechnol..

[29] Cai Y., Din Y., Ta G., Lia X. (2008). Production of 1,5-dihydroxy-3-methoxy-7-methylanthracene-9,10-dione by submerged culture of *Shiraia bambusicola*. J. Microbiol. Biotechnol..

[30] Yu J.H., Keller N. (2005). Regulation of secondary metabolism in filamentous fungi. Annu. Rev. Phytopathol..

[31] Arumugam G., Srinivasan S., Joshi G., Gopal D., Ramalingam K. (2015). Production and characterization of bioactive metabolites from piezotolerant deep sea fungus *Nigrospora* sp. in submerged fermentation. J. Appl. Microbiol..

[32] Netzker T., Fischer J., Weber J., Mattern D.J., König C.C., Valiante V., Schroeckh V., Brakhage A.A. (2015). Microbial communication leading to the activation of silent fungal secondary metabolite gene clusters. Front. Microbiol..

[33] Lang G., Wiese J., Schmaljohann R., Imhoff J.F. (2007). New pentaenes from the sponge-derived marine fungus *Penicillium rugulosum*: Structure determination and biosynthetic studies. Tetrahedron.

[34] Bringmann G., Gulder T.A., Lang G., Schmitt S., Stöhr R., Wiese J., Nagel K., Imhoff J.F. (2007). Large-scale biotechnological production of the antileukemic marine natural product sorbicillactone A. Mar. Drugs.

[35] Wiese J., Ohlendorf B., Blümel M., Schmaljohann R., Imhoff J.F. (2011). Phylogenetic identification of fungi isolated from the marine sponge *Tethya aurantium* and identification of their secondary metabolites. Mar. Drugs.

[36] Wang Y., Zheng J., Liu P., Wang W., Zhu W. (2011). Three new compounds from *Aspergillus terreus* pt06-2 grown in a high salt medium. Mar. Drugs.

[37] Varoglu M., Crews P. (2000). Biosynthetically diverse compounds from a saltwater culture of sponge-derived *Aspergillus niger*. J. Nat. Prod..

[38] Kobayashi M., Uehara H., Matsunami K., Aoki S., Kitagawa I. (1993). Trichoharzin, a new polyketide produced by the imperfect fungus *Trichoderma harzianum* separated from the marine sponge *Micale cecilia*. Tetrahedron Lett..

[39] Fouillaud M., Venkatachalam M., Llorente M., Magalon H., Cuet P., Dufossé L. (2017). Biodiversity of pigmented fungi isolated from marine environment in La Réunion island, Indian ocean: New resources for colored metabolites. J. Fungi.

[40] Venkatachalam M., Magalon H., Dufossé L., Fouillaud M. (2018). Production of pigments from the tropical marine-derived fungi *Talaromyces albobiverticillius*: New resources for natural red-colored metabolites. J. Food Compos. Anal..

[41] Venkatachalam M., Zelena M., Cacciola F., Ceslova L., Emmanuelle G.-V., Clerc P., Dugo P., Mondello L., Fouillaud M., Rotondo A. (2018). Partial characterization of the pigments produced by the marine-derived fungus *Talaromyces albobiverticillius* 30548. Towards a new fungal red colorant for the food industry. J. Food Compos. Anal..

[42] Overy D., Correa H., Roullier C., Chi W.C., Pang K.L., Rateb M., Ebel R., Shang Z., Capon R., Bills G. (2017). Does osmotic stress affect natural product expression in fungi?. Mar. Drugs.

[43] Huang J., Lu C., Qian X., Huang Y., Zheng Z., Shen Y. (2011). Effect of salinity on the growth, biological activity and secondary metabolites of some marine fungi. Acta Oceanol. Sin..

[44] Cho Y.J., Park J.P., Hwang H.J., Kim S.W., Choi J.W., Yun J.W. (2002). Production of red pigment by submerged culture of *Paecilomyces sinclairii*. Lett. Appl. Microbiol..

[45] Méndez A., Pérez C., Montañéz J.C., Martínez G., Aguilar C.N. (2011). Red pigment production by *Penicillium purpurogenum* GH2 is influenced by pH and temperature. J. Zhejiang Univ. Sci. B.

[46] Niknejad F., Moshfegh M., Najafzadeh M.J., Houbraken J., Rezaei S., Zarrini G., Faramarzi M.A., Nafissi-Varcheh N. (2013). Halotolerant ability and alpha-amylase activity of some saltwater fungal isolates. Iran. J. Pharm. Res..

[47] Velmurugan P., Lee Y.H., Nanthakumar K., Kamala-Kannan S., Dufossé L., Mapari S.A., Oh B.T. (2010). Water-soluble red pigments from *Isaria farinosa* and structural characterization of the main colored component. J. Basic Microbiol..

[48] Li Y.G., Zhang F., Wang Z.T., Hu Z.B. (2004). Identification and chemical profiling of monacolins in red yeast rice using high-performance liquid chromatography with photodiode array detector and mass spectrometry. J. Pharm. Biomed. Anal..

[49] Mapari S.A.S., Meyer A.S., Thrane U. (2006). Colorimetric characterization for comparative analysis of fungal pigments and natural food colorants. J. Agric. Food Chem..

[50] Ogbonna C.N. (2016). Production of food colorants by filamentous fungi. Afr. J. Microbiol. Res..

[51] Babitha S., Soccol C.R., Pandey A. (2007). Solid-state fermentation for the production of *Monascus* pigments from jackfruit seed. Bioresour. Technol..

[52] Aujla I.S., Paulitz T.C. (2017). An improved method for establishing accurate water potential levels at different temperatures in growth media. Front. Microbiol..

[53] Masuma R., Yamaguchi Y., Noumi M., Omura S., Namikoshi M. (2001). Effect of sea water concentration on hyphal growth and antimicrobial metabolite production in marine fungi. Mycoscience.

[54] Liu S., Li J., Wu Y., Ren Y., Liu Q., Wang Q., Zhou X., Cai M., Zhang Y. (2017). De novo transcriptome sequencing of marine-derived *Aspergillus glaucus* and comparative analysis of metabolic and developmental variations in response to salt stress. Genes Genom..

[55] Mapari S.A.S. (2009). Chemotaxonomic Exploration of Fungal Biodiversity for Polyketide Natural Food Colorants… Discovery & Evaluation of Cell Factories, and Characterization of Pigments. Ph.D. Thesis.

[56] Gunde-Cimerman N., Oren A., Plemenitaš A. (2005). Adaptation to Life at High Salt Concentrations in Archaea, Bacteria, and Eukarya.

[57] Ritchie D. (1957). Salinity optima for marine fungi affected by temperature. Am. J. Bot..

[58] Ritchie D. (1959). The efect of salinity and temperature on marine and other fungi from various climates. Bull. Torrey Bot. Club.

[59] Dunn P.H., Baker G.E. (1983). Filamentous fungi of the Psammon habitat at Enewetak atoll, Marshall islands. Mycologia.

[60] Janso J.E., Bernan V.S., Greenstein M., Bugni T.S., Ireland C.M. (2005). *Penicillium dravuni*, a new marine-derived species from an alga in fiji. Mycologia.

[61] Lorenz R., Molitoris H.-P. (1992). Combined influence of salinity and temperature (*Phoma*-pattern) on growth of marine fungi. Can. J. Bot..

[62] O’Mahony R.J., Burns A.T.H., Millam S., Hooley P., Fincham D.A. (2002). Isotropic growth of spores and salt tolerance in *Aspergillus nidulans*. Mycol. Res..

[63] Kunčič M.K., Kogej T., Drobne D., Gunde-Cimerman N. (2010). Morphological response of the halophilic fungal genus *Wallemia* to high salinity. Appl. Environ. Microbiol..

[64] Blomberg A., Adler L. (1992). Physiology of osmotolerance in fungi. Adv. Microb. Physiol..

[65] Bowman S.M., Free S.J. (2006). The structure and synthesis of the fungal cell wall. BioEssays.

[66] Kapteyn J.C., Van Den Ende H., Klis F.M. (1999). The contribution of cell wall proteins to the organization of the yeast cell wall. Biochim. Biophys. Acta Gen. Subj..

[67] Mager W.H., Siderius M. (2002). Novel insights into the osmotic stress response of yeast. FEMS Yeast Res..

[68] Castillo G., Demoulin V. (1997). Nacl salinity and temperature effects on growth of three wood-rotting basidiomycetes from a Papua New Guinea coastal forest. Mycol. Res..

[69] Santos-Ebinuma V.C., Roberto I.C., Teixeira M.F., Pessoa A. (2014). Improvement of submerged culture conditions to produce colorants by *Penicillium purpurogenum*. Braz. J. Microbiol..

[70] Chintapenta L.K., Rath C.C., Maringinti B., Ozbay G. (2014). Culture conditions for growth and pigment production of a mangrove *Penicillium* species. J. Multidiscip. Sci. Res..

[71] Sutherland I.W. (1990). Biotechnology of Microbial Exopolysaccharides.

[72] Hajjaj H., Blanc P., Groussac E., Goma G., Uribelarrea J., Loubiere P. (1999). Improvement of red pigment/citrinin production ratio as a function of environmental conditions by *Monascus ruber*. Biotechnol. Bioeng..

[73] Ahn J., Jung J., Hyung W., Haam S., Shin C. (2006). Enhancement of *Monascus* pigment production by the culture of *Monascus* sp. J101 at low temperature. Biotechnol. Prog..

[74] Chintapenta L.K., Rath C.C., Maringinti B., Ozbay G. (2014). Pigment production from a mangrove *Penicillium*. Afr. J. Biotechnol..

[75] Chadni Z., Rahaman M.H., Jerin I., Hoque K.M.F., Reza M.A. (2017). Extraction and optimisation of red pigment production as secondary metabolites from *Talaromyces verruculosus* and its potential use in textile industries. Mycology.

[76] Lopes F.C., Tichota D.M., Pereira J.Q., Segalin J., de Oliveira Rios A., Brandelli A. (2013). Pigment production by filamentous fungi on agro-industrial byproducts: An eco-friendly alternative. Appl. Biochem. Biotechnol..

[77] Řezanka T., Spížek J. (2005). Griseofulvin and other biologically active halogen containing compounds from fungi. Stud. Nat. Prod. Chem..

[78] Hsu Y.-W., Hsu L.-C., Liang Y.-H., Kuo Y.-H., Pan T.-M. (2011). New bioactive orange pigments with yellow fluorescence from *Monascus*-fermented *Dioscorea*. J. Agric. Food Chem..

[79] Eman M.M., Abbady M.S. (2014). Secondary metabolites and bioactivity of the *Monascus* pigments, review. Glob. J. Biotechnol. Biochem..

[80] Patakova P. (2013). *Monascus* secondary metabolites: Production and biological activity. J. Ind. Microbiol. Biotechnol..

[81] Martinkova L., Veselý D. (1995). Biological activity of polyketide pigments produced by the fungus *Monascus*. J. Appl. Microbiol..

